# Latent profile analysis of artificial intelligence anxiety among undergraduate nursing students: a cross-sectional study

**DOI:** 10.3389/fpubh.2026.1971125

**Published:** 2026-09-30

**Authors:** Jiali Xu, Xiaoqin Yu, Zhen Fang, Xiaoying Wang, Xianxian Xing, Juanjuan Cao, Xin Yang

**Affiliations:** 1 Jiujiang University Medical College, Jiujiang, China; 2 Department of Oncology, Southwest Jiaotong University, The Third People’s Hospital of Chengdu, Chengdu, Sichuan, China

**Keywords:** artificial intelligence anxiety, cross-sectional study, influencing factors, latent profile analysis, undergraduate nursing students

## Abstract

**Aim/objective:**

This study aimed to classify subgroups of artificial intelligence (AI) anxiety among nursing undergraduates using latent profile analysis, describe their demographic characteristics, and identify factors associated membership in each latent profile.

**Background:**

Artificial intelligence (AI) has been widely implemented in clinical nursing practice, but nursing undergraduates often experience AI anxiety, which impedes their adaptation to smart medical services. Few existing studies have stratified students according to anxiety levels to formulate targeted intervention measures.

**Design:**

A cross-sectional study.

**Methods:**

Participants were 526 nursing undergraduates recruited from several medical colleges in Jiangxi, Hunan, Hubei, and Sichuan provinces as well as Chongqing Municipality, China. Questionnaires, including a general information questionnaire, the Artificial Intelligence Anxiety Scale, and the Artificial Intelligence Self-Efficacy Questionnaire, were distributed. Mplus version 8.3 was used for latent profile analysis, and multinomial logistic regression was performed to analyze factors associated with profile membership.

**Results:**

A total of 526 students were included. The total AI anxiety score was 57.76 ± 15.94, indicating a moderate level of AI anxiety. AI anxiety among undergraduate nursing students was classified into three latent profiles: the low AI anxiety group (53.2%), moderate AI anxiety group (12.7%), and high AI anxiety group (34.0%). Multinomial logistic regression analysis showed that gender, participation in AI-related education or courses, attitude toward AI, and monthly household income (Renminbi [RMB]) were independently associated with membership in latent profiles among undergraduate nursing students with AI anxiety (*p* < 0.05).

**Conclusion:**

Nursing undergraduates present distinct heterogeneous patterns of AI anxiety. These findings may help identify students with elevated AI anxiety and inform individualized educational interventions.

## Introduction

With the rapid integration of artificial intelligence (AI) into clinical nursing, intelligent monitoring equipment, generative AI writing tools, and AI-powered clinical decision-making tools have been widely used in nursing education and patient treatment processes (1). To counteract the ongoing shift toward intelligent healthcare, the World Health Organization (WHO) has revised competency standards for the future nursing professionals, requiring nurses to be competent in basic AI operations and to learn how to think in collaboration with AI (2). As members of the reserve pool for the clinical nursing workforce, undergraduate nursing students constitute an important group who will be practicing intelligent nursing in the future. The psychological adaptation of nursing students to the use of AI technologies will undoubtedly affect their adaptability in the future clinical practice (3). The scientific evidence shows that AI literacy, which involves knowledge of AI technologies, practical skills, and ethical understanding, is an important professional competency among undergraduate nursing students (4). However, the widespread use of AI technologies has created psychological obstacles for nursing students. A significant number of undergraduate nursing students lack systematic education on AI technologies and therefore exhibit varying levels of AI anxiety when using intelligent nursing tools (5). AI anxiety refers to the negative emotional state involving fear, concern, and tension during the interaction with the AI technology. Such anxiety may reduce students’ motivation to learn about AI and further hinder the development of AI literacy (6).

Many studies conducted in China and other countries have examined the current status and factors influencing AI literacy and AI anxiety among undergraduate nursing students (7). Available cross-sectional survey studies have shown that there is moderate AI literacy among Chinese undergraduate nursing students, and that gender, family economic status, and attitudes toward AI training experience are major predictors of AI literacy (8). Different independent studies have shown that there is moderate AI anxiety among Chinese undergraduate nursing students, and there is a significant negative association between AI anxiety and AI literacy (9). Nevertheless, the majority of previous research employed variable-based statistical analyses to examine the two variables in question. This methodology might not capture heterogeneity within individuals’ AI anxiety and fails to examine the relationships between various subtypes of anxiety and levels of AI literacy (10). More importantly, there have been very few instances in which the latent profile analysis (LPA) technique, a person-centered analytic method, has been applied to detect distinct latent subtypes of AI anxiety. Differences in AI literacy, self-efficacy regarding AI, and demographic characteristics among anxiety subtypes have been compared (11). Bandura’s self-efficacy theory defines AI self-efficacy as the belief in one’s ability to accomplish AI-related tasks. Adequate self-efficacy can serve as a protective factor against AI anxiety and promote engagement in AI-based education (10). Tseng’s localized questionnaire for nursing students confirmed that AI self-efficacy is closely related to both AI literacy and AI anxiety. However, few LPA studies of AI anxiety have included self-efficacy as an associated variable for subgroup comparison (12).

The method of LPA categorizes participants into latent subgroups using homogeneous response patterns for several indicators of anxiety. In this way, it may uncover unobserved heterogeneity not captured by a single total score and generate findings that support the implementation of psychological interventions in stratified form (11). To address these research gaps, the cross-sectional design included recruiting undergraduate nursing students and using the four-dimensional AI anxiety scale to collect data. LPA was conducted to establish latent groups of AI anxiety. Differences between AI literacy and AI self-efficacy among anxiety profiles were then analyzed, and demographic, education, and AI-related factors associated with different AI anxiety profiles were also investigated (13). This study aimed to answer the following research questions: (1) What latent profiles of AI anxiety exist among undergraduate nursing students based on the four dimensions of learning anxiety, job replacement anxiety, sociotechnical blindness anxiety, and AI configuration anxiety? (2) What demographic and AI-related factors are associated with students’ latent anxiety profiles?

The study’s findings could provide an empirical basis for psychological interventions and curriculum development for AI in nursing education. Early detection of high-risk subtypes of AI anxiety in nursing students would enable tailored training and psychological interventions, help avoid negative attitudes toward AI technology, and promote higher AI self-efficacy and AI literacy among undergraduate nursing students. This would help the future nurses cope with the intelligent transformation of clinical nursing practice (14). The study also enriches the empirical literature on the psychological adaptation among undergraduate nursing students to AI. It develops a person-centered approach to analyzing such phenomena in the future. These findings have theoretical and practical implications for optimizing nursing education in the context of smart healthcare.

### Participants and sample size

From April to May 2026, a convenience sample of full-time undergraduate nursing students was recruited from several medical colleges across Jiangxi, Hunan, Hubei, and Sichuan provinces as well as Chongqing Municipality, central and southwestern China. The required sample size was estimated *a priori* using G*Power software (version 3.1.9.7) for multinomial logistic regression analysis. Given the exploratory nature of this study, a small effect size (f^2^ = 0.05), a significance level (α) of 0.05, and statistical power (1-β) of 0.95 were specified. There were 19 candidate predictors, including 9 questions from the general information survey, four dimensions of the Artificial Intelligence Anxiety Scale (AIAS), and 6 questions from the Artificial Intelligence Self-Efficacy Questionnaire (AISEQ). As an additional check, Kendall’s rule of thumb recommending 10 to 20 respondents per predictor was applied. With 19 predictors and a possible 20% invalid surveys, the required sample size ranged from 228 to 456 participants (15). As suggested by the results of the G*Power analysis, the minimal sample size required was 400 respondents. The final sample included 526 participants, satisfying both methodological criteria.

### Inclusion criteria

Undergraduate nursing students from several colleges and universities in China were recruited using convenience sampling. The inclusion criteria were as follows: (1) full-time undergraduate nursing students; (2) voluntary participation in the survey; and (3) ability to understand and complete the questionnaire independently.

### Exclusion criteria

The exclusion criteria were as follows: (1) interns, graduates, or students on long-term leave and (2) questionnaires with missing items or logical inconsistencies, which were considered invalid.

### Data collection

Data were collected online through the Wenjuanxing platform^1^ between April and May 2026. The tool used for data collection is a self-developed questionnaire. Informed consent was sought from the start of the survey and was coupled with pre-programmed skip logic such that any person refusing to give consent was redirected to the question submission page.

Before the formal survey, a pilot study was conducted with 10 randomly selected undergraduate nursing students to assess clarity, consistency, and the completion time of the items. It was evident that the questions posed to the students were clear, understandable, and unambiguous. The average completion time was about 6 min. This is evidence that the questionnaire was short and easy to use.

Information on the study purpose, inclusion and exclusion criteria, and anonymous questionnaire link was sent to the administrative departments of the participating nursing schools for distribution. To ensure data integrity, all items were required, and questionnaires with missing answers could not be submitted through the platform.

### Research tools

#### Demographic characteristics

The demographic part of this survey was created specifically for this research project by the researchers themselves. It included questions about respondents’ gender, grade in school, whether they had any background knowledge about AI or courses in AI, familiarity with AI tools, place of origin, per capita income of their families, readiness to choose nursing as a major, and their attitude toward AI.

#### AIAS

The AIAS, developed by Wang and Wang (16), includes 21 items across four dimensions: learning anxiety (8 items; anxiety about learning to understand and use AI technologies), job replacement anxiety (6 items; worry that AI will replace human jobs), sociotechnical blindness anxiety (4 items; concern that AI may distort human-machine relationships), and AI configuration anxiety (3 items; anxiety about operating, configuring, and maintaining AI systems). Each dimension score was computed as the sum of the corresponding items, and the total score was the sum of the four-dimensional scores. A 5-point Likert scale was used, ranging from 1 = not anxious at all to 5 = extremely anxious. Higher scores indicate higher levels of AI anxiety. The Cronbach’s α coefficient for the original scale was 0.915, and for the total scale in this study was 0.923. The Cronbach’s α coefficients of the four subscales in this sample were 0.473, 0.721, 0.807, and 0.869 for learning, job replacement, sociotechnical blindness, and AI configuration anxiety, respectively.

#### AI self-efficacy questionnaire

The AISEQ was used to assess AI self-efficacy among undergraduate nursing students. The scale was originally developed by Carolus (17), and its Chinese translation and cultural adaptation were completed by Tseng et al. (18). The questionnaire includes six items rated on a 5-point Likert scale, ranging from 1 = strongly disagree to 5 = strongly agree. Total scores range from 6 to 30, with higher scores indicating greater AI self-efficacy. In this study, the Cronbach’s α coefficient of the AISEQ was 0.900.

### Statistical analysis

Data were analyzed using Statistical Package for the Social Sciences (SPSS) version 27.0 and Mplus version 8.3. Categorical variables were described as frequencies and percentages, and between-group comparisons were performed using the *χ*^2^ test. Continuous variables were presented as the mean ± standard deviation (SD) or median and interquartile range, as appropriate. One-way analysis of variance (ANOVA) or the Kruskal-Wallis H test was used for between-group comparisons, depending on the data distribution.

LPA was performed in Mplus to classify distinct subgroups using the four AIAS dimension scores (learning anxiety, job replacement anxiety, sociotechnical blindness anxiety, and AI configuration anxiety) as continuous indicators. Models with one to four latent profiles were estimated and compared. Model fit was evaluated using the Akaike information criterion (AIC), Bayesian information criterion (BIC), sample-size-adjusted BIC (aBIC), entropy, Lo–Mendell–Rubin likelihood ratio test (LMR-LRT), and bootstrapped likelihood ratio test (BLRT). Lower AIC, BIC, and aBIC values indicated better model fit. Entropy values closer to 1 indicated greater classification accuracy, with values above 0.80 generally considered acceptable. A *p*-value < 0.05 for the LMR-LRT and BLRT indicated that the k-profile model fit the data significantly better than the (k − 1)-profile model. The final model was selected by jointly considering the statistical fit indices, classification accuracy, parsimony, and the interpretability of the resulting profiles. Classification accuracy of the selected model was further evaluated using the average posterior probabilities of class membership.

Multinomial logistic regression analysis was used to explore factors associated with different latent profiles of AI anxiety among undergraduate nursing students. The low AI anxiety profile served as the reference category of the outcome. Predictors were entered simultaneously, and multicollinearity was assessed using variance inflation factors (VIFs) and tolerance values. Odds ratios (ORs) with 95% confidence intervals (CIs) were reported as measures of effect size for all covariates, together with the reference category of each predictor. Model fit was evaluated using the likelihood-ratio *χ*^2^ test and Nagelkerke pseudo-R^2^. A two-tailed *p-*value < 0.05 was considered statistically significant.

### Research hypotheses

The purpose of this research is to determine the underlying profile of AI anxiety among undergraduate nursing students and the factors associated with it, thereby providing the necessary information to develop stratified interventions and optimize AI-related nursing education. The following hypotheses are generated:

*H1*: Multiple distinct latent profiles of AI anxiety exist among undergraduate nursing students based on the four dimensions of AI anxiety: learning anxiety, job replacement anxiety, sociotechnical blindness anxiety, and AI configuration anxiety.

*H2*: Demographic characteristics, AI-related learning and usage experiences, AI self-efficacy, and personal attitudes toward AI are significantly associated with latent profile membership for AI anxiety among undergraduate nursing students.

### Ethical considerations

All procedures involving human participants were conducted in accordance with the ethical standards of the institutional and/or national research committee and the 1964 Declaration of Helsinki and its later amendments, or comparable ethical standards. This study was approved by the Chengdu Third People’s Hospital Ethics Committee (approval number: 2026-S-195). Written informed consent was obtained from all participants.

## Results

### Demographic characteristics of undergraduate nursing students

In this study, 600 questionnaires were administered to undergraduate nursing students. Out of the total questionnaires returned, 526 were found to be valid and consequently analyzed, representing a response rate of 87.67%. Participants were 17–24-year old, with a mean age of 20.23 ± 1.02 years. Table 1 presents the frequency distribution of participants’ demographic characteristics.

**Table 1:** Frequency distribution of demographic characteristics among undergraduate nursing students (*n* = 526).

Variable	Number	Percent
Gender		
Male	112	21.29
Female	414	78.71
Grade		
Freshman	141	26.81
Sophomore	176	33.46
Junior	117	22.24
Senior	92	17.49
Family location		
Rural	153	29.09
City	373	70.91
Weekly internet usage time		
<2 h	33	6.27
2–5 h	62	11.79
5–8 h	128	24.33
>8 h	303	57.60
Participated in AI-related education/course		
Yes	359	68.25
No	167	31.75
Have used AI-related tools		
Yes	335	63.69
No	191	36.31
Monthly household income (RMB)		
<2,000	47	8.94
2,001-4,000	133	25.29
4,001-6,000	177	33.65
>6,000	169	32.13
Motivation for choosing nursing major		
Autoselect	285	54.18
Family	94	17.87
Other suggestions	90	17.11
Admission adjustment	57	10.84
Attitude toward AI		
Positive	248	47.15
Neutral	213	40.49
Negative	65	12.36

### AI anxiety and AI self-efficacy scores among undergraduate nursing students

Table 2 shows the AI Anxiety and AI Self-Efficacy scores of the surveyed undergraduate nursing students.

**Table 2:** AI anxiety and AI self-efficacy scores (*n* = 526, mean ± SD).

Variable	Mean ± SD	Item average score
Total score of AI anxiety	57.76 ± 15.94	2.75 ± 0.76
Learning anxiety	22.67 ± 5.06	2.83 ± 0.63
Job replacement anxiety	16.64 ± 5.23	2.74 ± 0.87
Sociotechnical-blindness anxiety	10.79 ± 4.26	2.70 ± 1.07
AI configuration anxiety	7.84 ± 3.34	2.61 ± 1.11
AI self-efficacy	22.52 ± 4.11	3.75 ± 0.69

### LPA and classification of AI anxiety

LPA was conducted using the four-dimensional scores of the AIAS as indicators. Table 3 provides the exact fit statistics of the competing one- to four-profile models. The information criteria (AIC, BIC, and aBIC) decreased continuously as the number of profiles increased, a pattern commonly observed as model complexity grows, and, therefore, could not, by itself, identify the optimal solution. Both the Lo–Mendell–Rubin adjusted likelihood ratio test and the bootstrap likelihood ratio test were statistically significant when moving from the one- to the two-profile model and from the two- to the three-profile model (all *p* < 0.001), indicating that each additional profile significantly improved model fit. The three-profile solution had the highest value of entropy (0.995), demonstrating excellent classification quality, and all three profiles had class probabilities well above 5% (0.532, 0.127, and 0.340 for the low, moderate, and high anxiety profiles, respectively), supporting stable classification and practical interpretability without extremely small subgroups. When a fourth profile was added, entropy declined to 0.924, the LMR-LRT was only marginally significant (= 0.024), and the additional profile mainly split the largest subgroup into two classes with similar score patterns, thereby reduced interpretability. After jointly evaluating the statistical fit indices, classification accuracy, parsimony, and interpretability, the three-profile model was retained as the final model of AI anxiety in nursing students.

**Table 3:** Comparison of latent profile model fit indices (*n* = 526).

model	AIC	BIC	aBIC	Entropy	LMRT	BLRT	Category probability
1	5,608.475	5,642.597	5,617.203	—	—	—	1.000
2	3,996.939	4,052.388	4,011.122	0.991	<0.001	<0.001	0.344/0.656
3	3,559.271	3,636.046	3,578.910	0.995	<0.001	<0.001	0.532/0.127/0.340
4	3,495.982	3,594.084	3,521.076	0.924	0.024	<0.001	0.532/0.127/0.171/0.169

The classification accuracy of the selected model was further supported by the average posterior probabilities of class membership shown in Table 4. The diagonal values were all ≥ 0.99 (range 0.990–1.000), indicating that participants were assigned to their most likely profile with very high certainty.

**Table 4:** Average posterior probabilities of class membership for the three-profile model (*n* = 526).

Most likely profile	Low AI anxiety (Profile 1)	Moderate AI anxiety (Profile 2)	High AI anxiety (Profile 3)
Low AI anxiety	1.000	0.000	0.000
Moderate AI anxiety	0.000	0.990	0.010
High AI anxiety	0.000	0.002	0.998

Average latent class probabilities for most likely latent class membership (obtained from the Mplus TECH11 output). Diagonal values ≥ 0.80 indicate adequate class separation.

The LPA results for AI anxiety among undergraduate nursing students are shown in Table 5 and Figure 1. Each profile was named according to its score pattern across the four dimensions: learning anxiety, job replacement anxiety, sociotechnical blindness anxiety, and AI configuration anxiety. Profile 1 included 280 participants (53.2% of the total sample), who had the lowest total and dimensional AI anxiety scores; this profile was labeled the low AI anxiety group. Profile 2 included 67 participants (12.7% of the total sample), whose scores on learning anxiety (19.40 ± 3.27), job replacement anxiety (12.85 ± 2.49), and sociotechnical blindness anxiety (8.36 ± 2.19) were comparable to those of the low-anxiety group (20.06 ± 3.31, 13.32 ± 2.74, and 8.07 ± 2.05, respectively). In contrast, their AI configuration anxiety (11.91 ± 0.45) approached the level observed in the high-anxiety group (11.89 ± 1.56). As the total AI anxiety score of this profile (52.61 ± 4.66) fell between those of the low (46.58 ± 5.28) and high (78.55 ± 5.90) anxiety profiles, it was labeled the moderate AI anxiety group; its defining characteristic was a dimension-specific elevation in anxiety about configuring and operating AI systems. Profile 3 included 179 participants (34.0% of the total sample), whose overall and individual scale scores were quite high compared with those of the other two groups, indicating serious worry about AI risks; thus, Profile 3 was referred to as the high AI anxiety group. From the line graph in Figure 1, clear hierarchies among the three groups are evident. The low anxiety group had very low scores in all dimensions, the high anxiety group had relatively high scores in all dimensions, and the moderate anxiety group showed a distinctive pattern with low scores on learning, job replacement, and sociotechnical blindness dimensions but a pronounced elevation on AI configuration anxiety. These patterns reflected differences in the internal structure of AI anxiety across the three groups.

**Table 5:** Mean scores and standard deviations for each dimension of the three-profile model of AI anxiety (*n* = 526).

Variable	Low AI anxiety	Moderate AI anxiety	High AI anxiety
Total score of AI anxiety	46.58 ± 5.28	52.61 ± 4.66	78.55 ± 5.90
Learning anxiety	20.06 ± 3.31	19.40 ± 3.27	27.98 ± 3.36
Job replacement anxiety	13.32 ± 2.74	12.85 ± 2.49	22.71 ± 2.62
Sociotechnical-blindness anxiety	8.07 ± 2.05	8.36 ± 2.19	15.97 ± 2.10
AI configuration anxiety	5.13 ± 0.99	11.91 ± 0.45	11.89 ± 1.56

**Figure 1:**
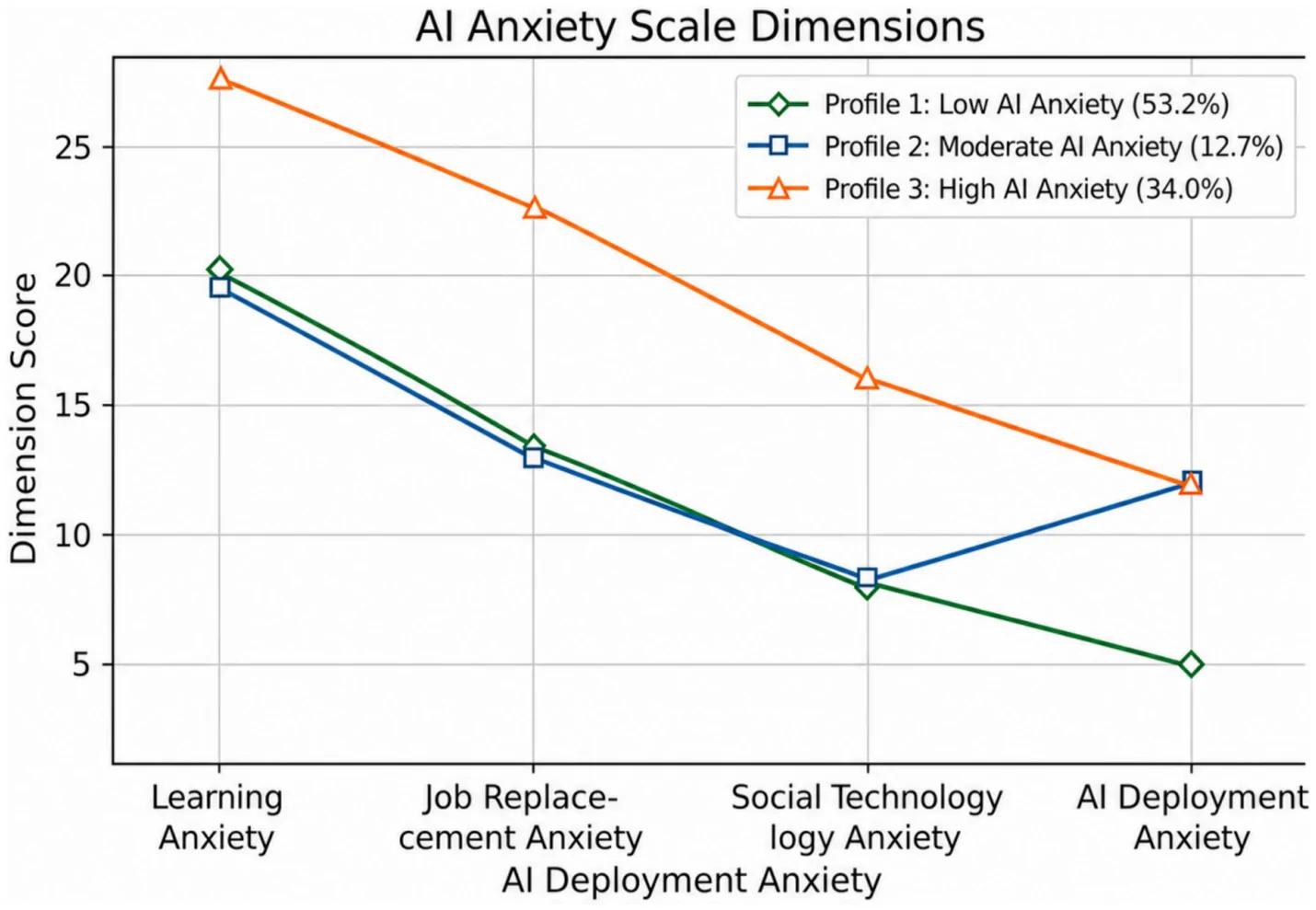
Distribution characteristics of latent profiles of AI anxiety among undergraduate nursing students.

### Univariate analysis of latent profiles of AI anxiety among undergraduate nursing students

As shown in Table 6, the high AI anxiety category consisted of 34.0% of the population and had a specific distribution of features in the group. These included a higher percentage of male participants, a higher number of people with no general knowledge of AI and no courses related to the subject, and a higher number of people with a per capita monthly family income of 4,000 Chinese Yuan (CNY). Overall, these findings suggest that students with less prior AI education and lower household income were overrepresented among those with the high AI anxiety profile.

**Table 6:** Comparison of sociodemographic characteristics across different AI anxiety profiles among undergraduate nursing students (*n* = 526).

Variable	Category	Low AI anxiety (*n* = 280)	Moderate AI anxiety (*n* = 67)	High AI anxiety (*n* = 179)	*χ^2^/F/H*	*p*
Gender	Male	35 (12.5)	32 (47.8%)	94 (52.5%)	92.950^a^	<0.001
	Female	245 (87.5)	35 (52.2%)	85 (47.5%)		
Grade	Freshman	74 (26.4%)	17 (25.4%)	50 (27.9%)	0.886^a^	0.990
	Sophomore	92 (32.9%)	24 (35.8%)	60 (33.5%)		
	Junior	62 (22.1%)	16 (23.9%)	39 (21.8%)		
	Senior	52 (18.6%)	10 (14.9%)	30 (16.8%)		
Rural city	Rural	87 (31.1%)	11 (16.4%)	55 (30.7%)	5.981^a^	0.050
	City	193 (68.9%)	56 (83.6%)	124 (69.3%)		
Participated in AI-related education/course	Yes	235 (83.9%)	32 (47.8%)	65 (36.3%)	114.126^a^	<0.001
	No	45 (16.1%)	35 (52.2%)	114 (63.7%)		
Have used AI-related tools	Yes	256 (91.4%)	64 (95.5%)	166 (92.7%)	1.335^a^	0.513
	No	24 (8.6%)	3 (4.5%)	13 (7.3%)		
Motivation for choosing nursing major	Autoselect	159 (56.8%)	37 (55.2%)	89 (49.7%)	11.035^a^	0.087
	Family	47 (16.8%)	16 (23.9%)	31 (17.3%)		
	Other Suggestions	41 (14.6%)	13 (19.4%)	36 (20.1%)		
	Admission adjustment	33 (11.8%)	1 (1.5%)	23 (12.8%)		
Monthly household income (RMB)	<2000	10 (3.6%)	16 (23.9%)	36 (20.1%)	115.812^a^	<0.001
	2001–4,000	50 (17.9%)	35 (52.2%)	75 (41.9%)		
	4,001–6,000	110 (39.3%)	10 (14.9%)	40 (22.3%)		
	>6,000	110 (39.3%)	6 (9.0%)	28 (15.6%)		
Weekly Internet Usage Time	≤2 h	18 (6.4%)	4 (6.0%)	11 (6.1%)	3.079^a^	0.799
	2–5 h	28 (10.0%)	7 (10.4%)	27 (15.1%)		
	5–8 h	68 (24.3%)	18 (26.9%)	42 (23.5%)		
	>8 h	166 (59.3%)	38 (56.7%)	99 (55.3%)		
Attitude toward AI	Positive	245 (87.5%)	18 (26.9%)	60 (33.5%)	175.520^a^	<0.001
	Neutral	30 (10.7%)	38 (56.7%)	85 (47.5%)		
	Negative	5 (1.8%)	11 (16.4%)	34 (19.0%)		

^a^Pearson χ^2^ test; ^b^Fisher exact test; *p* < 0.05 indicates statistical significance.

Whereas the majority of students who were identified in the high AI anxiety category already had some experience using AI technologies, it was evident that there were shortcomings on their part regarding systematic exposure to AI education and knowledge about AI, compared to students in the low AI anxiety category. This finding suggests that interaction with AI technologies alone may not be sufficient to alleviate anxiety about AI.

Statistically significant differences across latent profiles were observed in gender, prior exposure to AI-related knowledge or courses, per capita monthly household income, and attitudes toward AI (*p* < 0.001). The difference in place of origin was marginally statistically significant (*p* = 0.050). No statistically significant between-group differences were found in grade level, experience with AI tools, willingness to major in nursing, or weekly internet use duration (*p* > 0.05).

### Multicollinearity assessment

To evaluate the suitability of the selected explanatory and control variables for subsequent multinomial logistic regression analysis, variance inflation factors (VIFs) and tolerance values were calculated to assess multicollinearity. According to conventional cutoff criteria, a VIF below 5 and a tolerance value above 0.10 indicate no severe multicollinearity.

As shown in Table 7, the VIF values of all independent variables ranged from 1.011 to 1.477, all well below the common threshold of 5. The corresponding tolerance values ranged from 0.677 to 0.990, all exceeding 0.10. These results indicate that no obvious multicollinearity was present among the study variables. Among all predictors, attitude toward AI had the highest VIF of 1.477, with a tolerance of 0.677, whereas place of origin had the lowest VIF of 1.011 and the highest tolerance of 0.990. Given the low degree of collinearity among variables, all indicators were included in the subsequent multinomial logistic regression analysis.

**Table 7:** VIF analysis.

Variable	VIF	Tolerance
Gender	1.369	0.730
Grade	1.025	0.976
Rural city	1.011	0.990
Participated in AI-related education/course	1.395	0.717
Have used AI-related tools	1.037	0.964
Motivation for choosing nursing major	1.016	0.984
Monthly household income (RMB)	1.292	0.774
Weekly Internet usage time	1.032	0.969
Attitude toward AI	1.477	0.677

### Multinomial logistic regression analysis of latent profiles of AI anxiety

Latent profiles of AI anxiety were set as the dependent variable, with the low AI anxiety profile designated as the reference group. Variables showing statistically significant differences in the univariate analysis, including gender, prior exposure to AI-related knowledge or courses, per capita monthly household income, and attitudes toward AI, were entered simultaneously into the multinomial logistic regression model. The reference categories of the predictors were: female (gender), no participation in AI-related education/courses, monthly household income < 2,000 CNY, and negative attitude toward AI. Detailed coding assignments for all variables are presented in Table 8. The overall model was statistically significant (likelihood-ratio *χ*^2^ = 274.271, *df* = 14, *p* < 0.001; Nagelkerke pseudo-*R*^2^ = 0.475). ORs with their 95% CIs were reported as measures of effect size for all covariates.

**Table 8:** Coding method for independent variables.

Independent variable	Assignment method
Latent profile	Low AI anxiety profile = 1; Moderate AI anxiety profile = 2; High AI anxiety profile = 3 (reference: Low AI anxiety profile)
Gender (male)	Male = 1; Female = 0 (reference)
Participated in AI-related education/course (yes)	Yes = 1; No = 0 (reference)
Monthly household income (RMB) 2,001–4,000	2,001–4,000 CNY = 1; <2,000 CNY = 0 (reference)
Monthly household income (RMB) 4,001–6,000	4,001–6,000 CNY = 1; <2,000 CNY = 0 (reference)
Monthly household income (RMB) > 6,000	>6,000 CNY = 1; <2,000 CNY = 0 (reference)
Attitude toward AI: positive	Positive = 1; Negative = 0 (reference)
Attitude toward AI: Neutral	Neutral = 1; Negative = 0 (reference)

The multinomial logistic regression results showed that being male was independently associated with higher odds of membership in the moderate AI anxiety profile (OR = 2.355, *p* = 0.020, 95% CI = 1.143–4.851). A per capita monthly household income of 4,001–6,000 CNY (OR = 0.121, *p* < 0.001, 95% CI = 0.040–0.368), a monthly income above 6,000 CNY (OR = 0.084, *p* < 0.001, 95% CI = 0.025–0.287), and positive attitudes toward AI (OR = 0.103, *p* < 0.001, 95% CI = 0.030–0.358) were independently associated with lower odds of membership in the moderate AI anxiety profile.

Stronger associations were observed for the high AI anxiety profile. Male participants had significantly higher odds of membership in the high AI anxiety profile (OR = 2.838, *p* < 0.001, 95% CI = 1.615–4.988). In contrast, prior exposure to AI-related knowledge or courses (OR = 0.254, *p* < 0.001, 95% CI = 0.151–0.429), per capita monthly household income of 2,001–4,000 CNY (OR = 0.396, *p* = 0.048, 95% CI = 0.158–0.992), 4,001–6,000 CNY (OR = 0.225, *p* = 0.001, 95% CI = 0.090–0.561), and above 6,000 CNY (OR = 0.215, *p* = 0.001, 95% CI = 0.085–0.547), as well as positive attitudes toward AI (OR = 0.115, *p* < 0.001, 95% CI = 0.040–0.335), were independently associated with lower odds of membership in the high AI anxiety profile. All corresponding odds ratios were below 1, indicating that access to AI-related knowledge, higher household income, and positive technological attitudes were associated with lower odds of membership in the high AI anxiety profile. The full regression results are presented in Table 9.

**Table 9:** Results of the multinomial logistic regression analysis for latent profiles of AI anxiety among undergraduate nursing students (*n* = 526).

Comparison	Variables	*β*	SE	Wald *χ*^2^	*p*	OR (95% CI)
Moderate AI anxiety profile vs. Low AI anxiety profile	Intercept	1.738	0.740	5.517	0.019	5.685 (1.333 ~ 24.241)
	Gender (reference: Female)					
	Male	0.856	0.369	5.394	0.020	2.355 (1.143 ~ 4.851)
	Participated in AI-related education/course (reference: No)					
	Yes	−0.661	0.366	3.260	0.071	0.516 (0.252 ~ 1.058)
	Monthly household income (reference: <2,000 CNY)					
	2,001–4,000 CNY	−0.853	0.510	2.793	0.095	0.426 (0.157 ~ 1.159)
	4,001–6,000 CNY	−2.108	0.566	13.875	<0.001	0.121 (0.040 ~ 0.368)
	>6,000 CNY	−2.476	0.627	15.613	<0.001	0.084 (0.025 ~ 0.287)
	Attitude toward AI (reference: Negative)					
	Positive	−2.271	0.635	12.801	<0.001	0.103 (0.030 ~ 0.358)
	Neutral	−0.195	0.623	0.098	0.755	0.823 (0.243 ~ 2.790)
High AI anxiety profile vs. Low AI anxiety profile	Intercept	2.867	0.661	18.787	<0.001	17.583 (4.809 ~ 64.286)
	Gender (reference: Female)					
	Male	1.043	0.288	13.144	<0.001	2.838 (1.615 ~ 4.988)
	Participated in AI-related education/course (reference: No)					
	Yes	−1.369	0.267	26.272	<0.001	0.254 (0.151 ~ 0.429)
	Monthly household income (reference: <2,000 CNY)					
	2,001–4,000 CNY	−0.925	0.468	3.906	0.048	0.396 (0.158 ~ 0.992)
	4,001–6,000CNY	−1.490	0.466	10.237	0.001	0.225 (0.090 ~ 0.561)
	>6,000 CNY	−1.536	0.476	10.419	0.001	0.215 (0.085 ~ 0.547)
	Attitude toward AI (reference: Negative)					
	Positive	−2.161	0.544	15.773	<0.001	0.115 (0.040 ~ 0.335)
	Neutral	−0.523	0.564	0.860	0.354	0.593 (0.196 ~ 1.791)

SE, standard error; OR, odds ratio; 95% CI, 95% confidence interval; reference, reference group. The low AI anxiety profile was the reference category of the outcome; the reference category of each predictor is indicated in parentheses.

## Discussion

### Overall status of AI anxiety among undergraduate nursing students

This cross-sectional survey showed that undergraduate nursing students from central and southwestern China generally experienced a moderate level of AI anxiety, consistent with existing studies of nursing student cohorts in China (16). Among the four anxiety dimensions, learning anxiety and job replacement anxiety were the main psychological burdens, whereas concerns about AI equipment configuration in hospitals were relatively mild (19).

The prevalence of anxiety about job replacement suggests high levels of job insecurity amid the ongoing AI-driven revolution in nursing practice (20). Nursing students are constantly introduced to AI-enabled diagnosis and nursing robots in their classes and during clinical introductions, which might reinforce the notion that AI can take the place of bedside nursing (11). Learning anxiety, the second most prevalent dimension, might be linked to inadequacies in AI education in the nursing curriculum. Mandatory and systematic modules on AI are not always included in the curriculum; therefore, the learning materials may lack coherence, and the technical skills training may not be sufficient. The above-mentioned deficiencies could increase the difficulties students face in learning about AI (21). In contrast, undergraduate students are rarely involved in hospital-level digital infrastructure planning or equipment management during their studies. Therefore, they may perceive AI configuration risks less strongly than clinical nursing administrators (22–24).

### Latent profile heterogeneity of AI anxiety among undergraduate nursing students

Unlike the regression methods based on variables commonly used in previous studies, LPA was applied in the current study as an individual-based analysis to account for individual differences that may be overlooked by an aggregate-score approach; thereby filling an existing gap in the literature (25). Comprehensive evaluation of multiple model fit indices supported a three-profile classification scheme with excellent classification accuracy and a stable subgroup distribution (7).

These three categories of anxiety showed unique structures of anxiety rather than simply linear variations in level of score. The low AI anxiety group maintained relatively positive views toward AI-assisted learning and career development, without worrying about technological replacement or ease of operation (17, 26). Notably, the low-anxiety profile comprised the majority of the sample (53.2%). It was characterized by the most favorable AI-related characteristics observed in this study: 83.9% of these students had participated in AI-related education or courses, 87.5% held positive attitudes toward AI, and 78.6% came from households with a monthly income above 4,000 CNY (Table 6). This profile composition is consistent with prior evidence that systematic AI education and positive attitudes toward AI are associated with lower AI anxiety among nursing students (13, 16, 27), and with studies reporting inverse relationships among AI literacy, AI self-efficacy, and AI anxiety (23). Consistent with Bandura’s self-efficacy theory, students with adequate AI-related knowledge and positive attitudes may experience mastery in using AI tools and vicarious learning from successful examples, which could prevent anxiety from developing across all four dimensions and help them remain in the low-anxiety profile (10, 12). The predominance of this profile may also reflect the increasing availability of AI-related courses in Chinese nursing curricula and the growing digital familiarity of the current student generation (21, 24); however, these explanations remain tentative and should be examined in the future studies.

The moderate AI anxiety group (12.7%) was the smallest subgroup. Its defining feature was a dimension-specific pattern: scores on learning anxiety, job replacement anxiety, and sociotechnical blindness anxiety were comparable to those of the low-anxiety profile, whereas AI configuration anxiety (11.91 ± 0.45) approached the level of the high-anxiety profile (11.89 ± 1.56). This pattern suggests that, for a minority of students, AI anxiety is not a generalized negative state but a focused apprehension about operating, configuring, and maintaining AI systems in clinical settings. As the total AI anxiety score of this profile (52.61 ± 4.66) fell between those of the low- and high-anxiety profiles, the label “moderate AI anxiety” was retained; however, its interpretation should emphasize the dimension-specific configuration concern rather than a uniformly intermediate level of anxiety. Such a focused profile would not have been detectable by variable-centered analyses that aggregate all dimensions into a single total score (7, 10). As direct LPA comparisons for AI anxiety are scarce, whether this configuration-focused subgroup replicates in other samples and settings remains an open question for the future research. Students in this profile may be particularly sensitive to the practical aspects of AI implementation, such as equipment-operation errors or maintenance difficulties encountered during simulation training; however, the cross-sectional data cannot confirm this mechanism.

The high AI anxiety subgroup showed pervasive negative emotions across all four dimensions and faced combined pressure from technical learning, job replacement, sociotechnical risks, and equipment operation failures. This subgroup represents a population that may benefit the majority from prioritized psychological support and educational interventions (28, 29).

The variables gender, experience with AI, household income, and attitude toward AI turned out to be statistically different between the groups, while grade, usage of AI daily, reason for choosing the nursing program, and time spent online turned out not to be significantly associated with the profiles (30, 31). Notably, low daily usage of AI tools did not distinguish the anxiety profiles, which suggests that casual daily contact with AI alone may not be sufficient to alleviate deeply rooted negative attitudes toward it, and that systematic AI training may be more relevant to reducing AI-related anxiety (32, 33). Urban–rural origin reached only marginal statistical significance, suggesting that digital divide-related differences had limited influence on anxiety stratification in this sample (34).

### Independent factors associated with latent profiles of AI anxiety based on multinomial logistic regression

After accounting for multicollinearity, multinomial logistic regression further clarified the distinct protective and risk factors associated with the moderate- and high-anxiety profiles, supporting Hypothesis 2.

### Gender as an independent factor associated with profile membership

The male nursing students had increased odds of being members of moderate and high anxiety groups as compared to female students, which is different from what many previous studies indicated about higher anxiety levels among females because of AI (35, 36). The reason behind such a difference could be related to the gender makeup of the nursing program, where women are considered the majority. It may thus have more professional identification than men who may experience specific job-related pressure and an increased threat of being replaced by AI because of their low numbers in the female-dominated discipline (37, 38). These results suggest that gender-tailored psychological support and technical training may be worth exploring to address the concerns of male nursing students; however, this finding requires confirmation in the future studies.

### Participation in AI-related education/courses as a factor associated with lower odds of high anxiety

Participation in AI-related education or courses was associated with significantly lower odds of membership in the high AI anxiety profile. Still, the association with the moderate AI anxiety profile did not reach statistical significance. Formal AI training provides systematic theoretical knowledge and simulation practice in the context of AI. Such training might overcome any wrong assumptions about the replacement ability of AI by providing clear knowledge that AI acts as a support tool in nursing tasks (39). However, students without education on AI lack cognitive models to evaluate the pros and cons of using AI. Thus, they might be more likely to misunderstand negative data about the hazards of AI technology and become prone to having high anxiety profiles (40, 41). The lack of a significant association with the moderate anxiety profile may be explained by the specific anxiety structure of this group of students, because their anxiety was focused on the implementation and configuration of AI equipment in hospitals. This issue is not often covered in general AI courses. It can affect the effectiveness of these courses in reducing this specific type of anxiety (42, 43). This finding suggests that nursing schools should not only offer general AI literacy courses but also add modules on the deployment, maintenance, and clinical integration of hospital intelligent equipment to address students’ anxiety about equipment configuration (44).

### Monthly household income as a socioeconomic factor associated with profile membership

Higher monthly household income was associated with lower odds of membership in the moderate- and high-anxiety profiles. In comparison to their counterparts from low-income families, students from middle- and higher-income families were found to have significantly lower chances of being diagnosed with AI anxiety disorder. However, only income above 4,000 RMB showed a considerable protective influence against anxiety. Families from higher socio-economic backgrounds can provide continuous access to digital equipment, AI learning facilities, and technology-based learning practices, which help foster a more objective understanding of AI and reduce fear of the technology (34, 45). In contrast, learners who belong to poorer families may face the problem of not having sufficient access to digital information and a lack of formal education in AI, which would lead to the development of the phenomenon of “information gap anxiety” in relation to new medical applications of AI (2). Universities should therefore provide free AI workshops and open digital learning resources to reduce socioeconomic disparities in AI anxiety among students (26, 46, 47).

### Attitude toward AI as the factor with the strongest inverse association

Positive attitudes toward AI showed the strongest inverse association with membership in both the moderate- and high-anxiety profiles, whereas neutral perceptions showed no significant association (48). Students with positive attitudes may view AI as an auxiliary tool that reduces workload rather than as a career competitor. They may also be more likely to seek technical learning resources and develop a positive cognition–behavior–emotion cycle that buffers anxiety. Conversely, students with neutral or negative attitudes may overemphasize the ethical risks, privacy concerns, and technical limitations of AI, leading to avoidance of AI-related learning and persistent psychological stress (49, 50). Nursing educators should therefore prioritize the development of positive perceptions of medical AI through clinical case sharing in intelligent nursing, ethics seminars, and lectures on career prospects (51–53).

### Theoretical and practical implications

#### Theoretical implications

First, this study enriches empirical research on AI-related psychological adaptation among undergraduate nursing students by using person-centered LPA (54). This approach moves beyond the limitations of traditional variable-centered methods, which mainly examine overall anxiety scores and may overlook internal subgroup heterogeneity. The three distinct latent anxiety profiles identified in this study provide a new classification framework for the future research on AI-related psychological responses in nursing education. Second, this study applies person-centered LPA to distinguish heterogeneous anxiety subtypes and extends the application of Bandura’s self-efficacy theory to AI education in nursing (55) by explaining the general negative association between AI self-efficacy and AI anxiety. Third, multinomial logistic regression quantitatively clarified the stratified associations of demographic, educational, and attitudinal factors with different anxiety subtypes. These findings reveal distinct patterns in which the same variables are associated with distinct latent profiles and offer useful analytical perspectives for the future cross-sectional and longitudinal studies.

#### Practical educational implications

Based on the three latent anxiety subgroups identified in this study, nursing colleges can implement stratified and targeted intervention strategies.

The high AI anxiety group (34.0%) warrants prioritized support. Institutions of higher learning should focus their efforts on male students, students with no prior knowledge of AI, students from low-income backgrounds, and those with negative attitudes toward AI. A mandatory curriculum in AI literacy that covers the operation, clinical use, and ethics of AI needs to be developed. Development of forums or salons on AI development for male nursing students will help address their specific anxiety concerning replacement at work. Free training materials on AI, along with assistance in obtaining digital tools, should be provided to poor students. Clinical sessions on intelligent nursing should be arranged to foster a more positive attitude toward AI.

For those experiencing moderate levels of anxiety in relation to AI technologies (12.7%), the solutions should focus on specific modules dealing with the application, use, and maintenance of the AI technology at the hospital level since this anxiety is related to issues of equipment design. Administrators of digital nursing in clinical can be encouraged to give presentations on topics such as intelligent equipment design, human-equipment interaction, and hospital maintenance.

The intervention for the low AI-related anxiety group (53.2%) will have to be aimed at preserving the adaptation to AI that is currently in place. Interventions can include AI nursing innovation training camps, where students will be encouraged to participate in intelligent nursing design competitions. The students can further take on a mentorship role to help other students who suffer from AI anxiety get AI tools.

In addition, comprehensive optimization of the nursing curriculum is needed. AI knowledge should be integrated into core professional courses, theoretical teaching should be balanced with hands-on simulation training, and independent modules on AI ethics and medical data privacy should be added. A multilevel AI training system should be developed that combines general literacy, clinical application, and equipment management. Long-term psychological monitoring of students’ AI anxiety should also be implemented. The AI Anxiety Scale can be used regularly to screen high-risk subgroups and provide timely psychological guidance (56).

## Limitations

The research has several limitations. First, although participants were recruited from several medical colleges across Jiangxi, Hunan, Hubei, Sichuan, and Chongqing Municipality, a convenience sampling strategy was used, and the number and geographic distribution of participating colleges were limited. The sample therefore cannot be considered representative of all Chinese undergraduate nursing students, and the generalizability of the three-profile classification and its associated factors beyond these institutions is limited. The future research should use more representative, probability-based multi-center sampling across eastern, central, and western China to examine whether the latent classes are stable across populations. Second, the research design is cross-sectional and only allows for the examination of associations between variables; causality cannot be inferred. The identified factors should therefore be interpreted as correlates of profile membership rather than as causes of AI anxiety. Longitudinal studies and randomized controlled intervention trials need to be conducted to determine the causal impact of curriculum-based interventions on alleviating AI anxiety. Third, the study population comprised only undergraduate nursing students and excluded professional and graduate nurses. It is important to conduct the future comparative studies that include participants at different career levels to determine the developmental process of AI anxiety among nursing students in relation to professional practice. Fourth, all data were collected using self-reported online questionnaires, which may have introduced reporting bias due to social desirability and common-method variance, and no objective behavioral measures of AI use or anxiety were included. Fifth, the latent profiles were derived from a single sample and their labels reflect the patterns observed in this dataset; the classification should be validated in independent samples. The future mixed-methods studies combining questionnaire surveys with in-depth interviews could provide additional qualitative evidence on the psychological characteristics of different latent profiles.

## Conclusion

Undergraduate nursing students from central and southwestern China showed a moderate overall level of AI anxiety. Significant individual heterogeneity was observed in the structure of AI anxiety, which could be classified into three latent profiles with distinct dimensional score patterns: low, moderate, and high AI anxiety. Gender, participation in AI-related courses, monthly household income, and personal attitudes toward AI were independently associated with latent anxiety profile membership. Male gender, lack of systematic AI education, lower family income, and negative attitudes toward AI were associated with higher odds of belonging to the moderate- or high-anxiety profiles. In contrast, participation in AI-related education, higher household income, and positive attitudes toward AI were associated with lower odds of membership in the high-anxiety profile. Nursing educators may use these profiles to identify heterogeneous AI anxiety subgroups among students and implement stratified psychological interventions and differentiated AI curriculum design. These measures may improve students’ AI self-efficacy and overall AI literacy and help the future nursing workforce adapt to the intelligent transformation of clinical nursing.

## Data Availability

The raw data supporting the conclusions of this article will be made available by the authors, without undue reservation.

## Footnotes

1
https://www.wjx.cn
